# Psychosocial factors that distinguish between men and women who have suicidal thoughts and attempt suicide: findings from a national probability sample of adults

**DOI:** 10.1017/S0033291721005195

**Published:** 2023-05

**Authors:** Cara Richardson, Kathryn A. Robb, Sally McManus, Rory C. O'Connor

**Affiliations:** 1 Suicidal Behaviour Research Laboratory, Institute of Health & Wellbeing, University of Glasgow, Glasgow, UK; 2 Institute of Health & Wellbeing, University of Glasgow, Glasgow, UK; 3 School of Health Sciences, City, University of London, UK

**Keywords:** Females, ideation-to-action, males, risk factors, suicide risk

## Abstract

**Background:**

Previous research has highlighted the importance of understanding which psychosocial factors distinguish between those with suicide thoughts compared to those who attempt suicide. This study aims to investigate these distinguishing factors further within an ideation-to-action framework and to explore sex differences in suicide risk.

**Methods:**

Participants (*n* = 7546, aged 16+) were from the cross-sectional Adult Psychiatric Morbidity Survey (APMS; 2014) of England. Face-to-face and self-completion questionnaires assessed lifetime suicidal ideation, lifetime suicide attempts, demographic characteristics, life experiences, social support, health and mental illness. Multinomial logistic regression examined factors differentiating between those with suicidal ideation only and suicide attempt histories (with or without suicidal ideation) in men and women.

**Results:**

Overall men were less likely to report suicidal thoughts and attempts, compared to females. More factors differentiated between suicidal thoughts and attempts in women compared to in men; these included hospital admission for mental illness, below degree level qualifications, being single and childhood adversity. In men, factors which significantly differentiated between suicidal thoughts and attempts included self-report of professional diagnosis of mental illness and childhood adversity. Higher levels of social support were associated with being in the suicidal thoughts group *v.* in the attempts group in men.

**Conclusion:**

This study identified some key differences between men and women in factors associated with suicide attempts compared to suicidal thoughts. The findings support the use of the ideation-to-action framework to investigate sex differences in suicidal behaviour. Future research should examine the extent to which these factors are associated with suicide risk over time.

## Introduction

Suicide remains one of the leading causes of death worldwide, with one person taking their own life every 40 s (World Health Organization, 2019). The WHO estimates that for every person who has died by suicide approximately 20 people have attempted suicide (World Health Organization, 2014). Understanding the factors that may influence the transition from suicidal thoughts to attempts is crucial as it is estimated that almost 30% of people will act on their thoughts of suicide and approximately 60% of those will do so in the year of the first onset of suicidal ideation (Nock et al., 2008). In a recent review, Turecki et al. (2019) outlined the importance of understanding suicide risk factors but recognised the difficulties in disentangling the influence of these factors across the suicidal spectrum, from thoughts to acts of suicide.

The ideation-to-action framework is a recent framework that aims to help disentangle such risk factors. It postulates that the development of suicidal ideation and the transition to a suicide attempt should be understood as separate processes (Klonsky, Saffer, & Bryan, 2018). This is consistent with the predominant models such as the interpersonal theory (IPTS) (Joiner, 2007; Joiner, Van Orden, Witte, & Rudd, 2009), integrated motivational-volitional (IMV) model (O'Connor, (2011; O'Connor & Kirtley, 2018) and three-step theory (3ST) (Klonsky & May, 2015).

Of course, disentangling the influence of risk factors across the suicide spectrum is important in the identification and treatment of individuals at increased risk of dying by suicide, but there have been few attempts to investigate gender differences in this regard. For example, the time taken to transition from thinking about suicide to acting on these suicidal thoughts may be shorter in men compared to women (Schrijvers, Bollen, & Sabbe, 2012). Although, other authors have suggested that men may not act sooner on suicidal thoughts than women; rather it may be that they are less likely to disclose their suicidal thoughts and when they do, they may be less likely to be heard (Dahlen & Canetto, 2002) and have less social and emotional support available to them than women (Canetto, 2017). Gendered stigma in relation to suicidal behaviour is also relevant here: men may feel there is stigma in terms of them experiencing suicidal thoughts whereas women may feel stigma in regard to the act of taking their own life (Canetto, 1993, 2017; Dahlen & Canetto, 2002; Deluty, 1989; McAndrew & Garrison, 2007). The perpetration of these gendered stigmas may reinforce the gender paradox of suicide (Canetto & Sakinofsky, 1998). Although many factors will overlap across genders understanding the differential impact of risk factors will help inform a more tailored response in terms of treatment and prevention strategies

Furthermore, taking a gendered approach to understanding suicide risk is important because we know that risk varies as a function of gender (Turecki et al., 2019). The gender paradox in suicide is well-established and postulates that women are more likely to attempt suicide, but men are more likely to die by suicide (Canetto & Sakinofsky, 1998). Also, men who die by suicide outnumber women in almost every country in the world, except in the 15–19 years age group (Naghavi, 2019). However, there has yet to be a comprehensive systematic review of sex differences in risk factors for suicide or factors which differentiate between thoughts and attempts in men and women.

To address this dearth of evidence, the aim of this study was to further investigate the psychosocial factors that distinguish between those who think about suicide and those who attempt suicide and to explore sex differences in suicide risk.

## Methods

### Sample

This study was a secondary analysis of the Adult Psychiatric Morbidity Survey (APMS) 2014 (McManus, Bebbington, Jenkins, & Brugha, 2016; McManus et al., 2020). The APMS 2014 is the fourth in a series of surveys which reports the prevalence of both treated and untreated psychiatric disorder in England, in a sample of 7546 people aged 16 and over. There were 3058 (40.5%) men and 4488 (59.5%) women in this study. Each survey involved interviewing a large, stratified probability sample of the general population, covering people living in private households. Interviewers visited the address to identify private households with at least one resident aged 16 or over, one person per household was interviewed (if consent was given) to reduce the burden on the household and to ensure privacy for the interviews (Byron et al., 2016). Face-to-face and self-completion measures were employed. The response rate was 57% and those who did not take part either refused participation or contact could not be established (McManus et al., 2016, 2020). Those who did not participate were more likely to be younger and men and that this was addressed by calibration weighting designed to ensure the sample profile was representative of the English household population aged 16 years and over (McManus et al., 2016, 2020).

In the current study, first phase interviews were included, which involved an initial interview with the whole sample including self-reports of health service diagnosis. Full details of the methodology have been described elsewhere (McManus et al., 2016, 2020). NHS Digital granted permission for use of the data reported herein.

### Measures

A wide range of variables were included, spanning sociodemographic characteristics, life experiences, as well as indicators of physical and mental health. Demographic characteristics of the sample can be found in online Supplementary Appendices 1–5.

### Suicidal history

The following items related to suicidal thoughts and attempts were asked: ‘Have you ever thought of taking your life, even though you would not actually do it?’ and ‘Have you ever made an attempt to take your life, by taking an overdose of tablets or in some other way?’ (McManus et al., 2016, 2020). For the purposes of this study, a new variable was created which classified participants as either having no suicidal history, suicidal thoughts only or a suicide attempt(s) (with or without reported suicidal thoughts) history. Help-seeking following a suicide attempt was also included. Participants were asked whether they sought help from anyone, a friend, a family member, a neighbour, GP/family doctor, at hospital, someone else or a mental health professional. Descriptive statistics for the suicidal behaviour and help-seeking can be found in online Supplementary Appendices 3–4 and Table 1.

**Table 1.** Prevalence of self-reported lifetime suicidal thoughts and attempts by sex

	Prevalence *N* (%)	Sex differences OR (95% CI)
No suicidal history		Reference category
Men	2563 (83.8%)	
Women	3570 (79.5%)	
All	6133 (81.3%)	
Suicidal thoughts only		
Men	348 (11.4%)	0.83 (0.72–0.96)*
Women (reference category)	571 (12.9%)	
All	929 (12.3%)	
Suicidal attempts (with or without thoughts)		
Men	147 (4.8%)	0.61 (0.50–0.74)**
Women (reference category)	337 (7.5%)	
All	484 (6.4%)	

*p = 0.01; **p < 0.0001.

### Sociodemographic characteristics

The following sociodemographic variables were included sex (binary coded: male or female), *de-facto* marital status (same-sex couple, divorced or separated, widowed, single and married or cohabitating), age (continuous variable) and ethnicity (mixed/multiple ethnicities/other ethnic groups, Asian/Asian British, Black/African/Caribbean/Black British or White). Sex was binary coded (male or female) in the APMS dataset (McManus et al., 2016, 2020) as participants were only presented with a binary option, which likely corresponded with their sex. Therefore, the term sex is used in this paper, although given this is a survey participants were able to self-report as they choose. Ethnicity was based on those used in the latest Census and the Office for National Statistics (ONS) questions for use in national surveys (McManus et al., 2016, 2020). Employment status was coded as either employed (including working in a family business); unemployed (and therefore looking and available for work); or economically inactive (including those who are unable to work due to disability or illness, students, retired or looking after the home). Rurality was coded into three categories: village, hamlet and isolated dwellings; town and fringe; urban. Quintile of the indices of multiple deprivation (QIMD) is an area-level indicator with a low score indicating less deprivation and a high score indicating higher levels of deprivation (McLennan et al., 2019). Highest educational qualification was coded as: no qualifications, below university degree level qualifications (e.g. CSE/O Level/GCSE/A Levels/Higher Educational Qualification) and degree level qualification.

### Life experiences

Life events variables selected for this analysis were childhood adversity (assessed using 12 items) and trauma (assessed using 21 items) (McManus et al., 2016, 2020). Childhood adversity (minimum score: 0, maximum: 12) and trauma (minimum score: 0, maximum score: 21) were computed as continuous variables, summing the number of types of childhood adversity or trauma experienced. Examples of the childhood adversity items include before age 12 – ‘did not have a safe place to stay’ and before 12 – ‘were ill but no one took you to the doctor?’. The trauma variables included ‘experienced sexual abuse at any time of your life’ and ‘experienced being homeless at any time in your life’.

Social support was assessed using the seven-item Social Support Networks (IMSR) questionnaire (Brugha et al., 1987). All items began with the stem ‘people I know – ’ followed by: ‘do things to make me happy’ and ‘make me feel loved’. Participants could respond with: not true, partly true or certainly true. A continuous variable was computed for total social support score (minimum score: 0, maximum score: 21) by adding together each participant's scores on the seven social support items.

### Health

One question from the 12-item Short Form Survey (SF-12) examined ‘health in general’ (Ware & Gandek, 1998) with the response options: excellent, very good, good, fair or poor. Smoking history was recorded as never smoked or ever smoked. Multimorbidity was calculated as a continuous variable counting the number of health conditions participants reported anytime in adulthood. Thirty-six health conditions were covered, including cancer, diabetes, epilepsy/fits, migraine or frequent headaches, dementia or Alzheimer's disease, cataracts/eyesight problems, ear/hearing problems, stroke, heart attack/angina, high blood pressure, bronchitis/emphysema, asthma, stomach ulcer/digestive problems, liver problems, bladder problems/incontinence, infectious disease, arthritis, bowel/colon problems and skin problems. The minimum reported health conditions were 0 and the highest number of health conditions reported was 14.

### Mental health and wellbeing

Two questions assessed the lifetime presence of any eight common mental disorders (phobia, panic attacks, post-traumatic stress disorder, depression, post-natal depression, nervous breakdown, obsessive compulsive disorder and seasonal affective disorder). With one question covering whether the participant thinks they have had each disorder and the other whether the participant reports being told by a professional that they have it. This was coded as ‘not applicable’, ‘yes’ or ‘no’. Although, it is important to note that the words ‘mental illness’ are not used with participants, the question used was: ‘Now please look carefully at this card. Do you think that you have ever experienced any of these?’. A binary variable (yes/no) also classified whether participants reported having been admitted to hospital or a ward specialising in mental health (lifetime).

### Statistical analysis

Prevalence estimates were generated using frequencies and cross-tabulations. Binomial logistic regression analysis was conducted to determine the odds of reporting lifetime suicidal thoughts and attempts by comparing men and women (Table 1). Women were the reference category in this analysis.

Separate multinomial univariate logistic regression analyses were conducted for each of the variables to inform the selection of items for inclusion in the multivariate analyses. The focus of this analysis was on suicidal thoughts *v.* suicide attempts (online Supplementary Appendix 6). The following variables were included in the univariate analysis: age, marital status, ethnicity, education, employment, QIMD, rurality, health in general, multimorbidity, smoking history, self-diagnosis of mental illness, professional diagnosis of mental illness, admission to hospital or ward specialising in mental health, childhood adversity, trauma and social support. Suicidal thoughts were the reference category, thence the analysis did not include participants with no suicidal history. Then the file was split by sex and the analysis was repeated for men and women (online Supplementary Appendices 7 and 8).

Odds ratios (OR) and 95% CIs are reported. A risk factor was deemed to be significant if the *p* value was <0.01 to account for multiple comparisons. All analyses were conducted using SPSS 25.

## Results

### Suicidal history

Overall, 484 (6.4%) participants reported suicide attempts, 929 (12.3%) reported suicidal thoughts only and 6133 (81.3%) reported no suicidal history (Table 1). Among men, 147 (4.8%) reported suicide attempts, 348 (11.4%) reported suicidal thoughts only and 2563 (83.8%) reported no suicidal history (Table 1). Among women, 337 (7.5%) reported suicide attempts, 571 (12.9%) reported suicidal thoughts only and 3570 (79.5%) reported no suicidal history (Table 1).

In the binary logistic regression (Table 1) men were less likely to report suicidal thoughts only [OR (95% CI) = 0.86 (0.75–1.00), *p* < 0.05] and suicide attempts [OR (95% CI) = 0.62 (0.51–0.76), *p* < 0.0001) than women.

### Participant characteristics

There were 3058 (40.5%) men and 4488 (59.5%) women in this study. Participants in the 35–54 age group (relative to other age groups) accounted for the highest proportion of people in both the suicidal thoughts (*n* = 379, 5.0%) and suicide attempts (*n* = 194, 2.6%) groups. Compared to those in the other marital status categories, single people also accounted for most people in the suicidal thoughts (*n* = 186, 11.7%) and suicide attempts (*n* = 273, 17.2%) groups. The majority of participants were white (90.3%). The highest proportion in the suicidal thoughts group (*n* = 470, 6.2%) and suicide attempts group (*n* = 252, 3.3%) has below degree level qualifications. The majority of participants in both groups were employed: suicidal thoughts (*n* = 571, 7.6%) and suicide attempts (*n* = 227, 3.0%). Area level deprivation (QIMD) was fairly evenly distributed across the suicidal thoughts and attempts groups. The majority of participants lived in urban settings: suicidal thoughts (*n* = 761, 10.1%) and suicide attempts (*n* = 419, 5.6%). Full demographic characteristics, health and psychosocial factors by suicidal history and help-seeking following a suicide attempt for the overall sample and in men and women can be found in online Supplementary Appendices 1–5.

### Factors associated with suicide ideation *v.* attempts

The full table detailing the multivariate multinomial univariate logistic regression for variables distinguishing between suicidal thoughts and attempts (overall and in males and females) can be found in online Supplementary Appendices 7 and 8.

### Sex differences

The findings in Table 2 detail the differences in risk and protective factors for suicidal behaviour in men and women (see online Supplementary Appendices 7 and 8) for the full multivariate multinomial logistic regression of variables distinguishing between participants by suicidal history and sex. Ethnicity did not significantly differentiate between suicidal thoughts and attempts in males and females (online Supplementary Appendices 7 and 8). The age of respondents also did not significantly differentiate between suicidal thoughts and attempts in males and females, but older age was associated with lower levels of suicide attempts in women (online Supplementary Appendix 7) and low levels of thoughts and attempts in men (online Supplementary Appendix 8).

**Table 2.** Multivariate multinomial logistic regression of variables distinguishing between participants who reported suicidal thoughts *v*. those who reported suicide attempts by sex^a^

Model variables	Overall		Males		Females	
	Fully adjusted OR	*p* Value	Fully adjusted OR	*p* Value	Fully adjusted OR	*p* Value
*Sociodemographics*						
Age	0.71 (0.59–0.86)	<0.0001	–	–	–	–
Sex						
Male	0.73 (0.55–0.95)	0.02	–	–	–	–
Female (ref)	–	–	–	–	–	–
Marital status						
Divorced or separated	1.26 (0.90–1.77)	0.19	0.68 (0.38–1.23)	0.20	1.44 (0.96–2.15)	0.19
Widowed	0.89 (0.50–1.58)	0.69	0.91 (0.34–2.43)	0.85	0.65 (0.34–1.24)	0.19
Single	1.36 (1.01–1.84)	0.04	1.14 (0.73–1.79)	0.56	1.71 (1.18–2.46)	0.004
Married or cohabitating (ref)	–	–	–	–	–	–
Ethnicity						
Mixed/multiple ethnicities/other ethnic groups	–	–	–	–	–	–
Asian/Asian British	–	–	–	–	–	–
Black/African/Caribbean/Black British	–	–	–	–	–	–
White (ref)	–	–	–	–	–	–
Education						
No qualifications	2.43 (0.98–6.03)	0.06	3.28 (0.86–12.44)	0.08	1.35 (0.41–4.53)	0.62
Below degree level qualifications	1.63 (1.18–2.25)	0.003	1.36 (0.82–2.24)	0.24	1.90 (1.28–2.82)	0.001
Degree level qualification (ref)	–	–	–	–	–	–
Employment						
Economically inactive	0.98 (0.73–1.31)	0.87	1.21 (0.76, 1.94)	0.41	0.81 (0.57, 1.16)	0.25
Unemployed	0.92 (0.53–1.62)	0.78	1.51 (0.64, 3.59)	0.35	0.71 (0.35, 1.42)	0.33
In employment (ref)	–	–	–	–	–	–
QIMD						
34.17->87.80 most deprived	1.58 (1.03–2.42)	0.04	1.59 (0.83–3.06)	0.16	1.78 (1.06–3.00)	0.03
21.35->34.17	1.27 (0.82–1.95)	0.27	0.92 (0.47–1.77)	0.79	1.66 (0.98–2.81)	0.06
13.79->21.35	1.12 (0.73–1.73)	0.61	1.06 (0.55–2.03)	0.87	1.24 (0.72–2.11)	0.44
8.49->13.79	0.80 (0.50–1.25)	0.31	0.71 (0.36–1.39)	0.31	0.86 (0.50–1.51)	0.61
0.53->8.49 least deprived (ref)	–	–	–	–	–	–
Rurality						
Village, hamlet and isolated dwellings	0.77 (0.44–1.33)	0.35	0.94 (0.44–1.99)	0.87	0.68 (0.34–1.35)	0.27
Town and fringe	0.97 (0.65–1.46)	0.89	0.95 (0.51–1.79)	0.88	1.00 (0.61–1.64)	0.99
Urban (ref)	–	–	–	–	–	–
*Health*						
Current health in general (SF1)	0.92 (0.81–1.03)	0.16	0.97 (0.80–1.17)	0.76	0.90 (0.78–1.04)	0.15
Multimorbidity (since age 16)	1.10 (1.04–1.17)	0.001	1.06 (0.97–1.16)	0.22	1.07 (1.00–1.15)	0.04
Smoking history						
Ever smoked	0.77 (0.58–1.03)	0.07	1.01 (0.64–1.58)	0.98	0.70 (0.49–0.98)	0.04
Never smoked (ref)						
*Mental health and wellbeing*						
Self-diagnosis – self-report of having ever had any of 8 CMD						
Yes	0.60 (0.37–0.97)	0.04	0.43 (0.22–0.84)	0.01	0.71 (0.38–1.33)	0.28
No (ref)	–	–	–	–	–	–
Ever diagnosed with any of 8 CMD						
Yes	2.27 (1.54–3.37)	<0.0001	2.72 (1.48–5.00)	0.001	2.02 (1.25–3.26)	0.004
No (ref)	–	–	–	–	–	–
Ever admitted to hospital or ward specialising in mental health						
Yes	4.54 (2.93–7.03)	<0.0001	0.79 (0.32–1.97)	0.62	6.11 (3.40–10.98)	<0.0001
No (ref)	–	–	–	–	–	–
*Life experiences*						
Childhood adversity	1.23 (1.13–1.34)	<0.0001	1.28 (1.10–1.49)	0.001	1.25 (1.12–1.39)	<0.0001
Trauma	1.05 (1.01–1.10)	0.01	1.03 (0.97–1.10)	0.36	1.06 (1.004–1.12)	0.04
Social support score	0.98 (0.94–1.02)	0.25	0.91 (0.86–0.96)	0.001	1.02 (0.97–1.07)	0.53

a Suicidal thoughts were the reference category.

### Suicidal thoughts *v.* suicide attempts in women

#### Risk factors and context in women

Factors which significantly distinguished between suicidal thoughts to suicide attempts in women (Table 2 and online Supplementary Appendix 7), in the multivariate model, were hospital admission for mental illness [OR (95% CI) = 6.11 (3.40–10.98), *p* < 0.0001], self-report of professional diagnosis of mental illness [OR (95% CI) = 2.02 (1.25–3.26), *p* = 0.004], below degree level qualifications [OR (95% CI) = 1.90 (1.28–2.82), *p* = 0.001], being single [OR (95% CI) = 1.71 (1.18–2.46), *p* = 0.004] and childhood adversity [OR (95% CI) = 1.25 (1.12–1.39), *p* < 0.0001].

#### Protective factors in women

None of the factors distinguished between females in the suicidal thoughts and suicide attempts groups (Table 2 and online Supplementary Appendix 7).

### Suicidal thoughts *v.* suicide attempts in men

#### Risk factors and context in men

In the multivariate model, a self-reported professional diagnosis of mental illness [OR (95% CI) = 2.72 (1.48–5.00), *p* = 0.001] and childhood adversity [OR (95% CI) = 1.28 (1.10–1.49), *p* = 0.001] significantly differentiated between suicidal thoughts and attempts in men (Table 2 and online Supplementary Appendix 8).

#### Protective factors in men

Among men, higher levels of social support [OR (95% CI) = 0.91 (0.86–0.96), *p* = 0.001] were associated with a reduced odds of reporting a suicide attempt compared to those with suicidal thoughts only (Table 2 and online Supplementary Appendix 8).

### Post hoc analysis

In response to a comment from a reviewer, post-hoc analyses were conducted to examine sex differences in the childhood adversity and trauma variables (online Supplementary Appendix 9).

In regard to childhood adversity (before 18) men were more likely to have experienced ‘an adult in your life hit, beat, physically hurt you (other than smacking)’ [OR (95% CI) = 1.42 (1.23–1.63), *p* < 0.0001]. Women were more likely to experience ‘got scared or felt really bad because adult in your life called you names, said mean things to you, or said they didn't want you’ [OR (95% CI) = 0.72 (0.61–0.84), *p* < 0.0001].

Men were more likely to experience the following childhood adversity (before 12) variables: ‘went to school in clothes that were dirty, torn, didn't fit because no clean ones available’ [OR (95% CI) = 1.33 (1.19–1.50), *p* < 0.0001] and ‘went hungry because no one got your meals ready or there was no food in the home’ [OR (95% CI) = 1.33 (1.17–1.51), *p* < 0.0001]. There was no childhood adversity (before 12) that women were more likely to experience.

In regard to the trauma variables, men were more likely to experience: ‘serious illness or injury at any time in your life’ [OR (95% CI) = 1.54 (1.39–1.70), *p* < 0.0001], ‘serious assault to yourself at any time in your life’ [OR (95% CI) = 1.30 (1.09–1.56), *p* = 0.003], ‘being made redundant or sacked from your job at any time in your life’ [OR (95% CI) = 2.49 (2.25–2.74), *p* < 0.0001], ‘looking for work without success for more than 1 month at any time in your life’ [OR (95% CI) = 1.97 (1.77–2.20), *p* < 0.0001], ‘major financial crisis, equivalent to loss of 3 months income at any time in your life’ [OR (95% CI) = 1.71 (1.50–1.97), *p* < 0.0001], ‘trouble with police involving court appearance at any time in your life’ [OR (95% CI) = 5.43 (4.37–6.75), *p* < 0.0001], ‘time in prison on remand or serving a sentence at any time in your life’ [OR (95% CI) = 6.64 (4.03–10.95), *p* < 0.0001], ‘violence at work at any time in your life’ [OR (95% CI) = 2.47 (1.89–3.25), *p* < 0.0001] and ‘being expelled from school at any time in your life’ [OR (95% CI) = 1.88 (1.40–2.52), *p* < 0.0001].

Women were more likely to experience ‘separation due to marital difficulties, divorce or steady relationship breakdown at any time in your life’ [OR (95% CI) = 0.80 (0.72–0.88), *p* < 0.0001], ‘violence in the home at any time in your life’ [OR (95% CI) = 0.39 (0.32–0.47), *p* < 0.0001], ‘sexual abuse at any time in your life’ [OR (95% CI) = 0.28 (0.22–0.36), *p* < 0.0001] and ‘running away from home at any time in your life’ [OR (95% CI) = 0.72 (0.58–0.90), *p* = 0.003].

The new childhood adversity and trauma variables were then entered into the multivariate analysis investigating sex differences in factors associated with suicidal thoughts *v.* suicide attempts but none emerged as significant.

## Discussion

The research aims were met with significant sex differences identified, highlighting important risk, context and protective factors in those with a history of suicidal thoughts *v.* suicide attempts. Specifically, this study investigated the factors differentiating between individuals who had attempted suicide compared to those who had thought about suicide. Women reported more suicidal thoughts and attempts compared to men, consistent with the gender paradox of suicide (Canetto & Sakinofsky, 1998). More factors differentiated between suicidal thoughts and attempts in women and these included hospital admission for mental illness, below degree level qualifications, being single and childhood adversity. In men, factors which significantly differentiated between suicidal thoughts and attempts included self-report of professional diagnosis of mental illness and childhood adversity. Higher levels of social support seem to protect against suicide attempts in men, as those in suicidal thoughts group reported higher levels of social support than those who had attempted suicide.

Mental illness emerged as an important factor in this study, with a professional diagnosis having a higher odds ratio of distinguishing between suicidal thoughts and attempts in men relative to women. This may reflect men having a higher threshold of perceived severity of mental illness before they seek help, compared to females (Freeman et al., 2017) such that the former may only seek help when they are in crisis. Nonetheless, these findings suggest that those with a mental illness diagnosis are more at risk of acting on their thoughts of suicide compared to those without.

Men and women differed in their risk factor profiles. A history of hospitalisation for mental illness was a significant risk factor in women, which supports previous research recommending suicide risk evaluation after psychiatric discharge (Forte, Buscajoni, Fiorillo, Pompili, & Baldessarini, 2019; Vuagnat, Jollant, Abbar, Hawton, & Quantin, 2020; Walter et al., 2019). A higher proportion of women in this sample sought help from a hospital (78 women *v.* 31 men) or mental health professional (11 women *v.* 4 men) following a suicide attempt, which may also account for this finding. Level of education was only a significant risk factor in women, which could be understood as part of a larger picture of socioeconomic disparities and early life experiences (Lorant, Kapadia, Perelman, & the Demetriq study group, 2021). It is often theorised that female suicides are precipitated by interpersonal problems (e.g. relationship issues) and male suicides are more linked to impersonal problems like financial issues (Canetto, 2008; Kposowa, 2001). Although it is important to consider the impact of sociodemographic disadvantage on suicide risk in women, particularly as previous research has noted that employment is a protective factor for both men and women (Canetto, 2008). Considering the broader life circumstances of men and women may provide a more accurate portrayal of the factors contributing to their suicidal distress. Despite some gender differences, common features emerged across men and women: the pervasive impacts of mental illness, hospitalisation and adverse life experiences. Taken together, these findings highlight the importance of a personalised approach to suicide risk assessment and prevention (Graney et al., 2020).

This study has extended the extant literature on the differences between those who think about suicide and those who attempt suicide. The differences in risk profiles identified by men and women provide a deeper insight into what these participants have experienced (online Supplementary Appendix 9), with women more likely to experience relational trauma and violence or sexual abuse at home whereas males were more likely to experience violence at work specifically or in general and neglect. Historic risk factors such as childhood trauma have been studied previously (Burke, Ammerman, Knorr, Alloy, & McCloskey, 2018) consistent with many of the predominant theories of suicidal behaviour. Such existing theories can be used as a framework to understand the emergence of suicidal behaviour but more needs to be done to understand the application of such models to explain sex and gender differences in suicide. Indeed, it has been proposed in the fluid vulnerability theory that such pre-existing risk factors have a higher likelihood of differentiating between individuals who think about suicide and those who attempt suicide as these individuals can be described as having ‘chronic’ suicide risk which persists over time (Bryan & Rudd, 2016; Zatti et al., 2017). This is also consistent with acquired capability component of the IPTS (Joiner, 2007; Joiner et al., 2009), 3ST (Klonsky & May, 2015) and the pre-motivational stage of IMV model (O'Connor & Kirtley, 2018). In the present study, childhood adversity was a risk factor for both males and females, these findings reinforce the long-term effects of early childhood experiences which may also impact upon social, mental health and emotional outcomes in adulthood (Haahr-Pedersen et al., 2020).

Some protective factors also emerged from this study. Social support was only protective in men, with high levels being associated with a reduced likelihood of suicide attempts. This is consistent with previous research showing that men may benefit from community social support more than women (Šedivy, Podlogar, Kerr, & De Leo, 2017). This may further reinforce the fact that men benefit from feeling valued by their peers, that they have a positive impact on their life (Richardson, Dickson, Robb, & O'Connor, 2021).

### Strengths and limitations

This sample, the Adult Psychiatric Morbidity Survey, is large, nationally representative of the adult population in England, and is well suited to addressing our study aims. The APMS samples participants from the general population, rather than patient lists or established panel samples. This allows for the examination of the ‘treatment gap’ as it will include people with mental health problems but who are not actively involved in treatment. The sample is also well stratified in terms of area level deprivation and allows for a range of individuals to be included.

Participants' likelihood and willingness to report suicidal history can be affected by various factors including data collection methods (Turecki et al., 2019). The APMS 2014 dataset includes both self-report and interview administered surveys. It was decided in this study to analyse the self-report data on suicidal history as this method of data collection yields higher levels of suicide attempts and ideation as participants tend to feel more comfortable disclosing previous suicidal behaviour in self-report questions compared to face-to-face completion.

A limitation of this study, like other such research detailed by Nock, Kessler, and Franklin (2016) is that it is difficult to ascertain whether these risk factors influence the probability of the outcome variables (suicidal ideation or attempts) or whether these are consequences of the attempt itself. Also, due to the nature of this study design, some factors may have occurred after rather than before the attempt and it is difficult to ascertain whether both the outcome and risk factors are caused by another factor which has not been adjusted for in this dataset. This study also predominantly assesses distal risk factors measuring lifetime prevalence and was unable to include more specific risk factors such as access to lethal means and exposure to suicide which may have a more significant influence on the transition from thoughts to attempts. Nonetheless, the findings from this study are valuable for targeting groups in need. In addition, the cross-sectional nature of this study is a limitation as it means that we cannot comment directly on the extent to which these variables predict the transition from suicidal thoughts to attempts over time. In addition, the sample was predominantly white and there were a small number of same-sex couples which may influence the lack of significant findings across different ethnicities and sexualities (see online Supplementary Appendix 1 for more information). The data analysed in this paper are limited by having binary coded sex variables (male or female); as a result it was not possible to conduct analyses of different genders.

Finally, the suicide questions used in this dataset (‘Have you ever thought of taking your life, even though you would not actually do it?’ and ‘Have you ever made an attempt to take your life, by taking an overdose of tablets or in some other way?’) are potentially leading questions and may be gendered. For example, in Anglo culture self-poisoning as a suicide method can be stigmatised as it is viewed as feminine (Canetto, 2017; Canetto & Sakinofsky, 1998). In countries, like Britain, where male suicides outnumber female suicides there can be assumptions that suicide attempts are feminine, due to the notion of having ‘failed’ to take one's own life (Canetto, 2017; Canetto & Sakinofsky, 1998). Thence this may have affected the likelihood of men disclosing their previous suicidal behaviour.

### Knowledge gaps and directions for future research

An issue with researching factors which distinguish between suicidal thoughts and attempts is knowing when the transition from thoughts to action will occur or how this transition will happen (Bryan & Rudd, 2016). It is important to consider the timing of risk factor measurement to determine the exact impact of these variables (Bryan & Rudd, 2016), which is an important area for future research.

As we found here, and as Mars et al. (2019) also noted, the effect sizes of identified factors are often small and have not been replicated. As a result, it is unclear how robust some of the findings are and it demonstrates the need for further research. Also, future research should consider other risk factors that have not been investigated within the context of the ideation to action framework. For example, differences in the neural response to the threat of death, bodily harm or illness may differ in individuals who attempted suicide compared to those who thought about suicide. Previous research (Weinberg, May, Klonsky, Kotov, & Hajcak, 2017) has identified that this was blunted in those who have attempted suicide, which requires further examination.

Future research should also examine the impact of stigma on self-disclosure of mental illness and suicide in men and women, particularly as this study found that women were more likely to report suicidal thoughts and attempts. As well as understand the ways in which men and women feel comfortable talking about their suicidal history and mental illness to limit the reinforcement of this gendered stigma regarding the gender paradox of suicide (Canetto, 1993, 1997, 2008; Canetto & Sakinofsky, 1998; Deluty, 1989; McAndrew & Garrison, 2007).

## Conclusions

Distinguishing between suicidal ideation and suicide attempts is an area of both clinical and theoretical importance and this study has uncovered some important distinguishing factors. Sex differences were also examined in this study, as women reported more suicidal ideation and suicide attempts in the APMS dataset than men. The findings suggest that a history of hospitalisation for mental illness was associated with being in the suicide attempt group in both males and females, highlighting the potential need for monitoring of risk following discharge. The long-term impact of life experiences such as childhood adversity should also be considered as suicide risk factors. Future research should aim to build on these findings, particularly prospectively assessing the progression from ideation to attempts in real time.

## Supplementary material

For supplementary material accompanying this paper visit https://doi.org/10.1017/S0033291721005195. click here to view supplementary material
