# Supplementary material for: Psychosocial factors that distinguish between men and women who have suicidal thoughts and attempt suicide: findings from a national probability sample of adults
Source: Psychol Med. 2022 Jan 11;53(7):3133–41. doi: 10.1017/S0033291721005195 (PMC10235670; doi:10.1017/S0033291721005195)
Supplement: Supplementary file 1 [file S0033291721005195sup001.docx]

**Appendices**

Appendix 1 – Demographic characteristics, health and psychosocial factors by suicidal history

| (N (%) or M (SD)) | | | | |
| --- | --- | --- | --- | --- |
| Psychosocial Factor | **No Suicidal History** | | **Suicidal Thoughts** | **Suicide Attempts** |
| Sociodemographics | | | | |
| Age | 16-34: 1209 (16.0%)  35-54: 1901 (25.2%)  55-74: 1997 (26.5%)  75+: 1002 (13.3%) | 16-34: 243 (3.2%)  35-54: 379 (5.0%)  55-74: 280 (3.7%)  75+: 50 (0.7%) | | 16-34: 143 (1.9%)  35-54: 194 (2.6%)  55-74: 138 (1.8%)  75+: 10 (0.1%) |
| Marital Status |  |  | |  |
| Same-sex couple | 5 (83.3%) | 1 (16.7%) | | 0 (0%) |
| Divorced or separated | 702 (72.5%) | 113 (11.7%) | | 153 (15.8%) |
| Widowed | 764 (89.6%) | 23 (2.7%) | | 66 (7.7%) |
| Single | 1129 (71.1%) | 186 (11.7%) | | 273 (17.2%) |
| Married or cohabitating (ref) | 3533 (85.5%) | 161 (3.9%) | | 437 (10.6%) |
| Ethnicity |  |  | |  |
| Mixed/multiple ethnicities/other ethnic groups | 117 (77.5%) | 22 (14.6%) | | 12 (7.9%) |
| Asian/Asian British | 314 (88.0%) | 25 (7.0%) | | 18 (5.0%) |
| Black/African/Caribbean/black British | 158 (80.2%) | 28 (14.2%) | | 11 (5.6%) |
| White (ref) | 5518 (81.0%) | 853 (12.5%) | | 442 (6.5%) |
| Education |  |  | |  |
| No qualifications | 150 (2.0%) | 15 (0.2%) | | 10 (0.1%) |
| Below degree level qualifications | 2691 (35.7%) | 470 (6.2%) | | 252 (3.3%) |
| Degree level qualification (ref) | 1427 (18.9%) | 290 (3.8%) | | 75 (1.0%) |
| Employment |  |  | |  |
| Economically inactive | 2763 (36.6%) | 337 (4.5%) | | 232 (3.1%) |
| Unemployed | 148 (2.0%) | 44 (0.6%) | | 26 (0.3%) |
| In employment (ref) | 3198 (42.4%) | 571 (7.6%) | | 227 (3.0%) |
| QIMD |  |  | |  |
| 34.17 -> 87.80 most deprived | 1067 (14.1%) | 185 (2.5%) | | 170 (2.3%) |
| 21.35 -> 34.17 | 1155 (15.3%) | 188 (2.5%) | | 114 (1.5%) |
| 13.79->21.35 | 1283 (17.0%) | 190 (2.5%) | | 90 (1.2%) |
| 8.49 -> 13.79 | 1269 (16.8%) | 221 (3.9%) | | 60 (0.8%) |
| 0.53 -> 8.49 least deprived (ref) | 1335 (17.7%) | 168 (2.2%) | | 51 (0.7%) |
| Rurality |  |  | |  |
| Village, hamlet and isolated dwellings | 617 (8.2%) | 83 (1.1%) | | 20 (0.3%) |
| Town & fringe | 631 (8.4%) | 108 (1.4%) | | 46 (0.6%) |
| Urban (ref) | 4861 (64.4%) | 761 (10.1%) | | 419 (5.6%) |
| Health | | | | |
| General Health (SF1) | 3.49 (1.12) | 3.23 (1.15) | | 2.72 (1.25) |
| Multimorbidity | 2.40 (2.14) | 2.85 (2.29) | | 3.64 (2.61) |
| Smoking History |  |  | |  |
| Ever smoked | 2495 (33.1%) | 289 (3.8%) | | 95 (1.3%) |
| Never smoked (ref) | 3608 (47.8%) | 661 (8.8%) | | 387 (5.1%) |
| Mental Health and Wellbeing | | | | |
| Self-diagnosis – self report of having ever had any of 8 CMD |  |  | |  |
| Yes | 2310 (30.6%) | 755 (10.0%) | | 439 (5.8%) |
| No (ref) | 3796 (50.3%) | 196 (2.6%) | | 46 (0.6%) |
| Prof diagnosis – ever diagnosed with any of 8 CMD |  |  | |  |
| Yes | 1337 (17.7%) | 565 (7.5%) | | 400 (5.3%) |
| No (ref) | 4761 (63.1%) | 385 (5.1%) | | 84 (1.1%) |
| Ever admitted to hospital or ward specialising in mental health |  |  | |  |
| Yes | 75 (1.0%) | 34 (0.5%) | | 103 (1.4%) |
| No (ref) | 6034 (80.0%) | 918 (12.1%) | | 382 (5.1%) |
| Life Experiences | | | | |
| Childhood Adversity | .88 (.88) | 1.30 (1.12) | | 2.04 (1.79) |
| Trauma | 3.57 (2.32) | 5.27 (2.96) | | 6.81 (3.58) |
| Social Support | 20.11 (2.36) | 19.68 (2.52) | | 18.64 (3.67) |

Appendix 2 – Demographic characteristics, health, psychosocial factors and suicidal history by gender

| Gender Differences in Descriptive Statistics  (N (%)* or M (SD)) | | | | | | |
| --- | --- | --- | --- | --- | --- | --- |
| Psychosocial Factor | **No Suicidal History** | | **Suicidal Thoughts** | | **Suicide Attempts** | |
|  | **Males** | **Females** | **Males** | **Females** | **Males** | **Females** |
| Sociodemographics | | | | | | |
| Age | 16-34: 491 (16.1%)  35-54: 759 (24.8%)  55-74: 907 (29.7%)  75+: 398 (13.0%) | 16-34: 718 (16.0%)  35-54: 1142 (25.4%)  55-74: 1090 (24.3%)  75+: 604 (13.5%) | 16-34: 76 (2.5%)  35-54: 142 (4.6%)  55-74: 121 (4.0%)  75+: 17 (0.6%) | 16-34: 167 (3.7%)  35-54: 237 (5.3%)  55-74: 159 (3.5%)  75+: 33 (0.7%) | 16-34: 37 (1.2%)  35-54: 56 (1.8%)  55-74: 51 (1.7%)  75+: 3 (0.1%) | 16-34: 106 (22.1%)  35-54: 138 (33.8%)  55-74: 87 (1.9%)  75+: 7 (0.2%) |
| Marital Status |  |  |  |  |  |  |
| Same-sex couple | 0 (0%) | 5 (100%) | 1 (100%) | 0 (0%) | 0 (0%) | 0 (0%) |
| Divorced or separated | 250 (76.5%) | 452 (70.5%) | 22 (6.7%) | 91 (14.2%) | 55 (16.8%) | 98 (15.3%) |
| Widowed | 196 (91.6%) | 568 (88.9%) | 6 (2.8%) | 17 (2.7%) | 12 (5.6%) | 54 (8.5%) |
| Single | 528 (74.3%) | 601 (68.5%) | 65 (9.1%) | 121 (13.8%) | 118 (16.6%) | 155 (17.7%) |
| Married or cohabitating (ref) | 1589 (88.0%) | 1944 (83.6%) | 53 (2.9%) | 108 (4.6%) | 163 (9.0%) | 274 (11.8%) |
| Ethnicity |  |  |  |  |  |  |
| mixed/multiple ethnicities/other ethnic groups | 51 (89.5%) | 66 (70.2%) | 4 (7.0%) | 10 (10.6%) | 2 (3.5%) | 18 (19.1%) |
| Asian/Asian British | 138 (90.8%) | 176 (85.9%) | 7 (4.6%) | 11 (5.4%) | 7 (4.6%) | 18 (8.8%) |
| black/African/Caribbean/black British | 55 (82.1%) | 103 (79.2%) | 8 (11.9%) | 7 (5.4%) | 4 (6.0%) | 20 (15.4%) |
| white (ref) | 2312 (83.3%) | 3206 (79.4%) | 328 (11.8%) | 308 (7.6%) | 134 (4.8%) | 525 (13.0%) |
| Education |  |  |  |  |  |  |
| no qualifications | 72 (2.4%) | 78 (1.7%) | 4 (0.1%) | 11 (0.2%) | 5 (0.2%) | 5 (0.1%) |
| Below degree level qualifications | 1170 (38.3%) | 1521 (33.9%) | 186 (6.1%) | 284 (6.3%) | 72 (2.4%) | 180 (4.0%) |
| degree level qualification (ref) | 590 (19.3%) | 837 (18.6%) | 104 (3.4%) | 186 (4.1%) | 23 (0.8%) | 52 (1.2%) |
| Employment |  |  |  |  |  |  |
| economically inactive | 1052 (34.4%) | 1711 (38.1%) | 117 (3.8%) | 220 (4.9%) | 78 (2.6%) | 154 (3.4%) |
| unemployed | 75 (2.5%) | 73 (1.6%) | 14 (0.5%) | 30 (0.7%) | 10 (0.3%) | 16 (0.4%) |
| In employment (ref) | 1428 (46.7%) | 1770 (39.4%) | 225 (7.4%) | 346 (7.7%) | 59 (1.9%) | 168 (3.7%) |
| QIMD |  |  |  |  |  |  |
| 34.17 -> 87.80 most deprived | 431 (14.1%) | 636 (14.2%) | 68 (2.2%) | 117 (2.6%) | 54 (1.8%) | 116 (2.6%) |
| 21.35 -> 34.17 | 462 (15.1%) | 693 (15.4%) | 78 (2.6%) | 110 (2.5%) | 31 (1.0%) | 83 (1.8%) |
| 13.79->21.35 | 519 (17.0%) | 764 (17.0%) | 69 (2.3%) | 121 (2.7%) | 28 (0.9%) | 62 (1.4%) |
| 8.49 -> 13.79 | 537 (17.6%) | 732 (16.3%) | 82 (2.7%) | 139 (3.1%) | 17 (0.6%) | 43 (1.0%) |
| 0.53 -> 8.49 least deprived (ref) | 606 (19.8%) | 729 (16.2%) | 59 (1.9%) | 109 (2.4%) | 17 (0.6%) | 34 (0.8%) |
| Rurality |  |  |  |  |  |  |
| village, hamlet and isolated dwellings | 287 (9.4%) | 330 (7.4%) | 29 (0.9%) | 54 (1.2%) | 7 (0.2%) | 13 (0.3%) |
| town & fringe | 299 (9.8%) | 332 (7.4%) | 39 (1.3%) | 69 (1.5%) | 15 (0.5%) | 31 (0.7%) |
| urban (ref) | 1969 (64.4%) | 2892 (64.4%) | 288 (9.4%) | 473 (10.5%) | 125 (4.1%) | 294 (6,6%) |
| Health | | | | | | |
| General Health (SF1) | 3.48 (1.13) | 3.48 (1.12) | 3.17 (1.15) | 3.27 (1.14) | 2.60 (1.32) | 2.77 (1.22) |
| Multimorbidity | 2.29 (2.10) | 2.49 (2.17) | 2.76 (2.29) | 2.90 (2.29) | 3.54 (2.57) | 3.69 (2.62) |
| Smoking History |  |  |  |  |  |  |
| ever smoked | 864 (28.3%) | 1631 (36.3%) | 92 (3.0%) | 197 (4.4%) | 27 (0.9%) | 68 (1.5%) |
| never smoked (ref) | 1689 (55.2%) | 1919 (42.8%) | 262 (8.6%) | 399 (8.9%) | 118 (3.9%) | 269 (6.0%) |
| Mental Health and Wellbeing | | | | | | |
| Self-diagnosis – self report of having ever had any of 8 CMD |  |  |  |  |  |  |
| yes | 755 (24.7%) | 1555 (34.6%) | 263 (8.6%) | 492 (11.0%) | 126 (4.1%) | 313 (7.0%) |
| no (ref) | 1799 (58.8%) | 1997 (44.5%) | 93 (3.0%) | 103 (2.3%) | 21 (0.7%) | 25 (0.6%) |
| Prof diagnosis – ever diagnosed with any of 8 CMD |  |  |  |  |  |  |
| yes | 371 (12.1%) | 966 (21.5%) | 184 (6.0%) | 381 (8.5%) | 114 (3.7%) | 286 (6.4%) |
| no (ref) | 2180 (71.3%) | 2581 (57.5%) | 171 (5.6%) | 214 (4.8%) | 33 (1.1%) | 51 (1.1%) |
| Ever admitted to hospital or ward specialising in mental health |  |  |  |  |  |  |
| yes | 31 (1.0%) | 44 (1.0%) | 17 (0.6%) | 17 (0.4%) | 33 (1.1%) | 70 (1.6%) |
| no (ref) | 2524 (82.5%) | 3510 (78.2%) | 339 (11.1%) | 579 (12.9%) | 114 (3.7%) | 268 (6.0%) |
| Life Experiences | | | | | | |
| Childhood Adversity | .83 (.89) | .91 (.88) | 1.24 (1.08) | 1.34 (1.14) | 1.85 (1.70) | 2.12 (1.83) |
| Trauma | 3.85 (2.47) | 3.37 (2.19) | 5.80 (3.10) | 4.94 (2.83) | 7.18 (3.68) | 6.64 (3.54) |
| Social Support | 19.89 (2.50) | 20.27 (2.24) | 19.33 (2.83) | 19.89 (2.30) | 17.29 (4.24) | 19.23 (3.22) |

Appendix 3 – Suicidal thoughts and behaviours

|  | Prevalence  N (%) | Sex Differences  OR (95% CI) |
| --- | --- | --- |
| Missing Data/Refused to Answer/Don’t Know |  |  |
| Men | 206 (6.7%) |  |
| Women (reference category) | 340 (7.6%) |  |
| All | 546 (7.2%) |  |
| No Suicidal History |  |  |
| Men | 2357 (77.1%) | Reference category |
| Women | 3230 (71.0%) |  |
| All | 5587 (75.0%) |  |
| Suicidal Thoughts and Attempts |  |  |
| Men | 142 (4.6%) | .62 [.51, .76]*** |
| Women (reference category) | 314 (7.0%) |  |
| All | 456 (6.0%) |  |
| Suicidal Thoughts (No Attempts) |  |  |
| Men | 348 (11.4%) | .82 [.71, .95]** |
| Women (reference category) | 581 (12.9%) |  |
| All | 929 (12.3%) |  |
| Suicide Attempt(s) (No Thoughts) |  |  |
| Men | 5 (0.2%) | .30 [.11, .79]* |
| Women (reference category) | 23 (0.5%) |  |
| All | 28 (0.4%) |  |

Appendix 4 – Help-seeking following a suicide attempt in males and females

| Help-seeking  Tried to get help from: | Number (%) | | | |
| --- | --- | --- | --- | --- |
|  | **Males** | | **Females** | |
|  | **Not mentioned** | **Mentioned** | **Not mentioned** | **Mentioned** |
| Anyone | 73 (50.0%) | 73 (50%) | 181 (53.7%) | 156 (46.3%) |
| A friend | 54 (74.0%) | 19 (26.0%) | 150 (77.3%) | 41 (22.7%) |
| A family member | 55 (75.3%) | 18 (24.7%) | 139 (76.8%) | 42 (23.2%) |
| A neighbour | 71 (97.3%) | 2 (2.7%) | 176 (97.2%) | 5 (2.8%) |
| GP/Family doctor | 33 (45.2%) | 40 (54.8%) | 90 (49.7%) | 91 (50.3%) |
| A hospital | 42 (57.5%) | 31 (42.5%) | 103 (56.9%) | 78 (43.1%)4 |
| Someone else | 72 (98.6%) | 1 (1.4%) | 181 (100%) | 0 (0%) |
| Mental health professional | 69 (94.5%) | 4 (5.5%) | 170 (93.9%) | 11 (6.1%) |
| Helpline number/support group | 72 (98.6%) | 1 (1.4%) | 178 (98.3%) | 3 (1.7%) |
| Other | 71 (97.3%) | 2 (2.7%) | 178 (98.3%) | 3 (1.7%) |

Appendix 5 – Gender Differences in Mental Illness Variables

| Mental Illness | Men  N (%) | | Women  N (%) | |
| --- | --- | --- | --- | --- |
|  | **Mentioned** | **Not mentioned** | **Mentioned** | **Not mentioned** |
| Self-diagnosed | | | | |
| Depression | 811 (26.5%) | 2246 (73.5%) | 1467 (32.7%) | 3018 (67.3%) |
| Post-Natal Depression | 2 (0.1%) | 3055 (99.9%) | 464 (10.3%) | 4021 (89.7%) |
| Nervous Breakdown | 129 (4.2%) | 2928 (95.8%) | 220 (4.9%) | 4265 (95.1%) |
| Obsessive Compulsive Disorder | 115 (3.8%) | 2942 (96.2%) | 162 (3.6%) | 4323 (96.4%) |
| Seasonal Affective Disorder | 103 (3.4%) | 2954 (96.6%) | 218 (4.9%) | 4267 (95.1%) |
| Panic Attacks | 408 (13.3%) | 2649 (86.7%) | 1104 (24.6%) | 3381 (75.4%) |
| Phobia | 217 (7.1%) | 2840 (92.9%) | 443 (9.9%) | 4042 (90.1%) |
| Post-Traumatic Stress Disorder | 94 (3.1%) | 2963 (96.9%) | 180 (4.0%) | 4305 (96.0%) |
| Professional diagnosis | | | | |
| Depression | 556 (18.2%) | 2499 (81.8%) | 1196 (26.7%) | 3284 (73.3%) |
| Post-Natal Depression | 0 (0%) | 3057 (100%) | 340 (7.6%) | 4144 (92.4%) |
| Nervous Breakdown | 92 (3.0%) | 2962 (97.0%) | 158 (3.5%) | 4325 (96.5%) |
| Obsessive Compulsive Disorder | 29 (0.9%) | 3028 (99.1%) | 57 (1.3%) | 4428 (98.7%) |
| Seasonal Affective Disorder | 15 (0.5%) | 3042 (99.5%) | 40 (0.9%) | 4444 (99.1%) |
| Panic Attacks | 217 (7.1%) | 2839 (92.9%) | 685 (15.3%) | 3795 (84.7%) |
| Phobia | 31 (1.0%) | 3025 (99.0%) | 66 (1.5%) | 4418 (98.5%) |
| Post-Traumatic Stress Disorder | 53 (1.7%) | 3004 (98.3%) | 106 (2.4%) | 4378 (97.6%) |

Appendix 6 – Multinomial logistic regression of demographic characteristics, health and psychosocial factors variables associated with suicidal history group membership

| Full Ideation to Action Model | | | | | | | | | | | | |
| --- | --- | --- | --- | --- | --- | --- | --- | --- | --- | --- | --- | --- |
| Model Variables | **Suicidal Ideation vs No Suicidal History*** | | | | **Suicide Attempts vs No Suicidal History**** | | | | **Suicidal Thoughts vs Suicide Attempts**** | | | |
|  | **Unadjusted OR** | **P value** | **Fully Adjusted OR** | **P value** | **Unadjusted OR** | **P value** | **Fully Adjusted OR** | **P value** | **Unadjusted OR** | **P value** | **Fully Adjusted OR** | **P value** |
| Sociodemographics | | | | | | | | | | | | |
| Age | .71 [.66, .77] | <.0001 | .72 [.64, .80] | <.0001 | .63 [.57, .69] | <.0001 | .51 [.43, .61] | <.0001 | .88 [.78, .99] | .03 | .71 [.59, .86] | <.0001 |
| Sex |  |  |  |  |  |  |  |  |  |  |  |  |
| male | .83 [.72, .96] | .01 | .99 [.84, 1.17] | .94 | .61 [.50, .74] | <.0001 | .71 [.55, .91] | .008 | .83 [.72, .96] | .01 | .73 [.55, .95] | .02 |
| female (ref) |  |  |  |  |  |  |  |  |  |  |  |  |
| Marital Status |  |  |  |  |  |  |  |  |  |  |  |  |
| Same-sex couple | - | - | - | - | 4.39 [.51, 37.78] | .18 | .30 [.02, 5.01] | .40 | - | - | - | - |
| Divorced or separated | 1.76 [1.44, 2.16] | <.0001 | 1.26 [1.00, 1.58] | .05 | 3.53 [2.74, 4.55] | <.0001 | 1.55 [1.12, 2.13] | .007 | 2.01 [1.48, 2.71] | <.0001 | 1.26 [.90, 1.77] | .19 |
| Widowed | .70 [.53, .92] | .009 | 1.34 [.97, 1.84] | .07 | .66 [.42, 1.03] | .07 | 1.16 [.67, 1.98] | .60 | .95 [.57, 1.57] | .83 | .89 [.50, 1.58] | .69 |
| Single | 1.96 [1.66, 2.31] | <.0001 | 1.61 [1.32, 1.95] | <.0001 | 3.62 [2.90, 4.51] | <.0001 | 2.23 [1.68, 2.96] | <.0001 | 1.85 [1.43, 2.40] | <.0001 | 1.36 [1.01, 1.84] | .04 |
| Married or cohabitating (ref) |  |  |  |  |  |  |  |  |  |  |  |  |
| Ethnicity |  |  |  |  |  |  |  |  |  |  |  |  |
| mixed/multiple ethnicities/other ethnic groups | 1.22 [.77, 1.93] | .41 | 1.27 [.76, 2.12] | .36 | 1.28 [.70, 2.34] | .42 | 1.26 [.60, 2.67] | .54 | 1.05 [.52, 2.15] | .89 | - | - |
| Asian/Asian British | .52 [.34, .78] | .002 | .76 [.48, 1.19] | .23 | .72 [.44, 1.16] | .18 | 1.42 [.77, 2.62] | .26 | 1.39 [.75, 2.57] | .30 | - | - |
| black/African/Caribbean/black British | 1.15 [.76, 1.72] | .51 | .95 [.59, 1.53] | .85 | .87 [.47, 1.61] | .66 | .52 [.25, 1.11] | .09 | .76 [.37, 1.54] | .44 | - | - |
| white (ref) |  |  |  |  |  |  |  |  |  |  |  |  |
| Education |  |  |  |  |  |  |  |  |  |  |  |  |
| no qualifications | .47 [.27, .82] | .008 | .63 [.34, 1.15] | .13 | 1.27 [.64, 2.50] | .50 | 1.53 [.67, 3.49] | .31 | 2.70 [ 1.15, 6.31] | .02 | 2.43 [.98, 6.03] | .06 |
| Below degree level qualifications | .86 [.73, 1.01] | .07 | .71 [.59, .85] | <.0001 | 1.78 [1.37, 2.34] | <.0001 | 1.17 [.85, 1.59] | .34 | 2.07 [1.54, 2.79] | <.0001 | 1.63 [1.18, 2.25] | .003 |
| 3 - degree level qualification (ref) |  |  |  |  |  |  |  |  |  |  |  |  |
| Employment |  |  |  |  |  |  |  |  |  |  |  |  |
| economically inactive | .69 [.60, .80] | <.0001 | .85 [.70, 1.02] | .09 | 1.18 [.98, 1.43] | .09 | .81 [.62, 1.08] | .15 | 1.71 [1.36, 2.15] | <.0001 | .98 [.73, 1.31] | .87 |
| unemployed | 1.62 [1.14, 2.31] | .008 | 1.11 [.74, 1.65] | .62 | 2.45 [1.58, 3.80] | <.0001 | 1.01 [.59, 1.74] | .96 | 1.52 [.91, 2.53] | .11 | .92 [.53, 1.62] | .78 |
| In employment (ref) |  |  |  |  |  |  |  |  |  |  |  |  |
| QIMD |  |  |  |  |  |  |  |  |  |  |  |  |
| 34.17 -> 87.80 most deprived | 1.36 [1.09, 1.70] | .008 | .94 [.73, 1.23] | .67 | 4.13 [2.99, 5.71] | <.0001 | 1.53 [1.03, 2.28] | .04 | 3.04 [2.08, 4.43] | <.0001 | 1.58 [1.03, 2.42] | .04 |
| 21.35 -> 34.17 | 1.30 [1.04, 1.62] | .02 | 1.02 [.79, 1.32] | .87 | 2.58 [1.84, 3.63] | <.0001 | 1.31 [.87, 1.96] | .20 | 2.00 [1.35, 2.95] | .001 | 1.27 [.82, 1.95] | .27 |
| 13.79->21.35 | 1.13 [.90, 1.41] | .29 | .95 [.74, 1.22] | .70 | 1.83 [1.28, 2.60] | .001 | 1.06 [.71, 1.61] | .77 | 1.62 [1.08, 2.42] | .02 | 1.12 [.73, 1.73] | .61 |
| 8.49 -> 13.79 | 1.36 [1.09, 1.69] | .006 | 1.25 [.99, 1.59] | .06 | 1.23 [.84, 1.81] | .28 | .99 [.64, 1.53] | .97 | .91 [.59, 1.39] | .66 | .80 [.50, 1.25] | .31 |
| 0.53 -> 8.49 least deprived (ref) |  |  |  |  |  |  |  |  |  |  |  |  |
| Rurality |  |  |  |  |  |  |  |  |  |  |  |  |
| village, hamlet and isolated dwellings | .85 [.66, 1.09] | .19 | .95 [.72, 1.25] | .70 | .38 [.24, .60] | <.0001 | .75 [.44, 1.27] | .29 | .44 [.27, .74] | .002 | .77 [.44, 1.33] | .35 |
| town & fringe | 1.11 [.90, 1.34] | .34 | 1.21 [.94, 1.55] | .14 | .85 [.62, 1.17] | .31 | 1.19 [.80, 1.76] | .39 | .76 [.53, 1.10] | .15 | .97 [.65, 1.46] | .89 |
| urban (ref) |  |  |  |  |  |  |  |  |  |  |  |  |
| Health | | | | | | | | | | | | |
|  |  |  |  |  |  |  |  |  |  |  |  |  |
| Health in general (SF1) | .83 [.78, .88] | <.0001 | .89 [.83, .97] | .005 | .57 [.53, .62] | <.0001 | .83 [.74, .92] | .001 | .70 [.63, .76] | <.0001 | .92 [.81, 1.03] | .16 |
| Multimorbidity | 1.09 [1.06, 1.13] | <.0001 | 1.03 [.99, 1.07] | .18 | 1.23 [1.19, 1.28] | <.0001 | 1.14 [1.08, 1.20] | <.0001 | 1.13 [1.08, 1.18] | <.0001 | 1.10 [1.04, 1.17] | .001 |
| Smoking History |  |  |  |  |  |  |  |  |  |  |  |  |
| ever smoked | .63 [.55, .74] | <.0001 | .84 [.71, 1.00] | .04 | .36 [.28, .45] | <.0001 | .63 [.48, .83] | .001 | .56 [.43, .73] | <.0001 | .77 [.58, 1.03] | .07 |
| never smoked (ref) |  |  |  |  |  |  |  |  |  |  |  |  |
| Mental Health and Wellbeing | | | | | | | | | | | | |
| Self-diagnosis – self report of having ever had any of 8 CMD |  |  |  |  |  |  |  |  |  |  |  |  |
| yes | 6.68 [5.64, 7.92] | <.0001 | 3.17 [2.53, 3.97] | <.0001 | 15.63 [11.48, 21.27] | <.0001 | 1.91 [1.21, 3.01] | .006 | 2.34 [1.66, 3.30] | <.0001 | .60 [.37, .97] | .04 |
| no (ref) |  |  |  |  |  |  |  |  |  |  |  |  |
| Prof diagnosis – ever diagnosed with any of 8 CMD |  |  |  |  |  |  |  |  |  |  |  |  |
| yes | 5.28 [4.57, 6.11] | <.0001 | 1.92 [1.58, 2.35] | <.0001 | 16.82 [13.19, 21.45] | <.0001 | 4.36 [3.00, 6.34] | <.0001 | 3.18 [2.43, 4.17] | <.0001 | 2.27 [1.54, 3.37] | <.0001 |
| no (ref) |  |  |  |  |  |  |  |  |  |  |  |  |
| Ever admitted to hospital or ward specialising in mental health |  |  |  |  |  |  |  |  |  |  |  |  |
| yes | 3.07 [2.03, 4.63] | <.0001 | 1.17 [.75, 1.82] | .48 | 21.84 [15.94, 29.92] | <.0001 | 5.24 [3.53, 7.78] | <.0001 | 7.12 [4.74, 10.68] | <.0001 | 4.54 [2.93, 7.03] | <.0001 |
| no (ref) |  |  |  |  |  |  |  |  |  |  |  |  |
| Life Experiences | | | | | | | | | | | | |
| Childhood Adversity | 1.52 [1.43, 1.62] | <.0001 | 1.23 [1.14, 1.32] | <.0001 | 2.10 [1.96, 2.26] | <.0001 | 1.61 [1.39, 1.65] | <.0001 | 1.38 [1.28, 1.49] | <.0001 | 1.23 [1.13, 1.34] | <.0001 |
| Trauma | 1.28 [1.25, 1.31] | <.0001 | 1.16 [1.13, 1.20] | <.0001 | 1.47 [1.42, 1.51] | <.0001 | 1.23 [1.18, 1.28] | <.0001 | 1.15 [1.11, 1.19] | <.0001 | 1.05 [1.01, 1.10] | .01 |
| Social Support Score | .94 [.91, .96] | <.0001 | .95 [.92, .99] | .004 | .87 [.85, .89] | <.0001 | .93 [.89, .97] | <.0001 | .93 [.90, .95] | <.0001 | .98 [.94, 1.02] | .25 |

*Reference category: no suicidal history

**Reference category: suicidal thoughts

Appendix 7 – Multinomial logistic regression of demographic characteristics, health and psychosocial factors associated with suicidal history in females

| Full Ideation to Action Model in Females | | | | | | | | | | | | |
| --- | --- | --- | --- | --- | --- | --- | --- | --- | --- | --- | --- | --- |
| Model Variables | **Suicidal Ideation vs No Suicidal History*** | | | | **Suicide Attempts vs No Suicidal History*** | | | | **Suicidal Thoughts vs Suicide Attempts**** | | | |
|  | **Unadjusted OR** | **P value** | **Fully Adjusted OR** | **P value** | **Unadjusted OR** | **P value** | **Fully Adjusted OR** | **P value** | **Unadjusted OR** | **P value** | **Fully Adjusted OR** | **P value** |
| Sociodemographics | | | | | | | | | | | | |
| Age | .69 [.63, .76] | <.0001 | .69 [.59, .80] | <.0001 | .61 [.54, .69] | <.0001 | .50 [.41, .63] | <.0001 | .88 [.76, 1.01] | .08 | - | - |
| Marital Status |  |  |  |  |  |  |  |  |  |  |  |  |
| Same-sex couple | - | - | - | - | - | - | - | - | - | - | - | - |
| Divorced or separated | 1.54 [1.20, 1.98] | .001 | 1.18 [.88, 1.57].27 |  | 3.62 [2.69, 4.88] | <.0001 | 1.81 [1.24, 2.65] | .002 | 2.36 [1.64, 3.38] | <.0001 | 1.44 [.96, 2.15] | .19 |
| Widowed | .68 [.50, .92] | .01 | 1.37 [.94, 1.99] | .10 | .54 [.32, .91] | .02 | 1.08 [.57, 2.04] | .82 | .80 [.44, 1.44] | .45 | .65 [.34, 1.24] | .19 |
| Single | 1.83 [1.47, 2.27] | <.0001 | 1.43 [1.11, 1.85] | .006 | 3.62 [2.75, 4.77] | <.0001 | 2.27 [1.60, 3.24] | <.0001 | 1.98 [1.43, 2.74] | <.0001 | 1.71 [1.18, 2.46] | .004 |
| Married or cohabitating (ref) |  |  |  |  |  |  |  |  |  |  |  |  |
| Ethnicity |  |  |  |  |  |  |  |  |  |  |  |  |
| mixed/multiple ethnicities/other ethnic groups | 1.67 [.98, 2.83] | .06 | 1.62 [.90, 2.94] | .11 | 1.58 [.80, 3.10] | .19 | 1.40 [.60, 3.28] | .43 | .95 [.43, 2.08] | .95 | - | - |
| Asian/Asian British | .63 [.38, 1.02] | .06 | .88 [.51, 1.52] | .64 | .65 [.35, 1.21] | .17 | 1.30 [.59, 2.86] | .51 | 1.04 [.49, 2.23] | .92 | - | - |
| black/African/Caribbean/black British | 1.19 [.73, 1.93] | .49 | 1.06 [.60, 1.88] | .84 | .71 [.33, 1.54] | .38 | .44 [.17, 1.14] | .09 | .60 [.25, 1.43] | .25 | - | - |
| white (ref) |  |  |  |  |  |  |  |  |  |  |  |  |
| Education |  |  |  |  |  |  |  |  |  |  |  |  |
| no qualifications | .59 [.30, 1.16] | .13 | .94 [.44, 2.00] | .87 | 1.03 [.40, 2.64] | .96 | 1.40 [.45, 4.38] | .56 | 1.74 [.57, 5.32] | .33 | 1.35 [.41, 4.53] | .62 |
| Below degree level qualifications | .85 [.69, 1.04] | .11 | .70 [.56, .88] | .003 | 1.91 [1.39, 2.63] | <.0001 | 1.31 [.90, 2.93] | .16 | 2.25 [1.57, 3.23] | <.0001 | 1.90 [1.28, 2.82] | .001 |
| degree level qualification (ref) |  |  |  |  |  |  |  |  |  |  |  |  |
| Employment |  |  |  |  |  |  |  |  |  |  |  |  |
| economically inactive | .65 [.54, .78] | <.0001 | .89 [.71, 1.13] | .35 | .94 [.75, 1.18] | .61 | .74 [.53, 1.04] | .08 | 1.44 [1.09, 1.91] | .01 | .81 [.57, 1.16] | .25 |
| unemployed | 1.96 [1.25, 3.08] | .003 | 1.59 [.95, 2.65] | .08 | 2.26 [1.29, 3.96] | .005 | 1.11 [.55, 2.21] | .78 | 1.15 [.61, 2.19] | .66 | .71 [.35, 1.42] | .33 |
| In employment (ref) |  |  |  |  |  |  |  |  |  |  |  |  |
| QIMD |  |  |  |  |  |  |  |  |  |  |  |  |
| 34.17 -> 87.80 most deprived | 1.22 [.92, 1.62] | .18 | .88 [.63, 1.23] | .46 | 3.86 [2.60, 5.75] | <.0001 | 1.55 [.95, 2.54] | .08 | 3.18 [2.00, 5.05] | <.0001 | 1.78 [1.06, 3.00] | .03 |
| 21.35 -> 34.17 | 1.05 [.79, 1.40] | .74 | .90 [.65, 1.24] | .52 | 2.56 [1.70, 3.87] | <.0001 | 1.49 [.91, 2.44] | .12 | 2.44 [1.51, 3.95] | <.0001 | 1.66 [.98, 2.81] | .06 |
| 13.79->21.35 | 1.01 [.76, 1.34] | .95 | .89 [.65, 1.21] | .45 | 1.73 [1.12, 2.66] | .01 | 1.06 [.64, 1.76] | .82 | 1.71 [1.05, 2.81] | .03 | 1.24 [.72, 2.11] | .44 |
| 8.49 -> 13.79 | 1.24 [.94, 1.63] | .12 | 1.16 [.86, 1.57] | .33 | 1.25 [.79, 1.99] | .34 | 1.00 [.59, 1.71] | .99 | 1.01 [.60, 1.70] | .97 | .86 [.50, 1.51] | .61 |
| 0.53 -> 8.49 least deprived (ref) |  |  |  |  |  |  |  |  |  |  |  |  |
| Rurality |  |  |  |  |  |  |  |  |  |  |  |  |
| village, hamlet and isolated dwellings | .96 [.71, 1.31] | .81 | 1.02 [.72, 1.45] | .90 | .39 [.22, .68] | .001 | .72 [.37, 1.40] | .33 | .40 [.22, .75] | .004 | .68 [.34, 1.35] | .27 |
| town & fringe | 1.28 [.97, 1.70] | .08 | 1.39 [1.02, 1.90] | .04 | .92 [.63, 1.36] | .68 | 1.39 [.86, 2.25] | .18 | .72 [.46, 1.13] | .15 | 1.00 [.61, 1.64] | .99 |
| urban (ref) |  |  |  |  |  |  |  |  |  |  |  |  |
| Health | | | | | | | | | | | | |
| Health in general (SF1) | .85 [.79, .92] | <.0001 | .91 [.83, 1.01] | .07 | .59 [.54, .65] | <.0001 | .82 [.71, .94] | .005 | .70 [.62, .78] | <.0001 | .90 [.78, 1.04] | .15 |
| Multimorbidity | 1.08 [1.04, 1.13] | <.0001 | 1.03 [.98, 1.09] | .23 | 1.22 [1.17, 1.28] | <.0001 | 1.14 [1.07, 1.22] | <.0001 | 1.13 [1.07, 1.19] | <.0001 | 1.07 [1.00, 1.15] | .04 |
| Smoking History |  |  |  |  |  |  |  |  |  |  |  |  |
| ever smoked | .59 [.49, .71] | <.0001 | .82 [.66, 1.00] | .05 | .30 [.23, .39] | <.0001 | .58 [.42, .81] | .001 | .51 [.37, .70] | <.0001 | .70 [.49, .98] | .04 |
| never smoked (ref) |  |  |  |  |  |  |  |  |  |  |  |  |
| Mental Health and Wellbeing | | | | | | | | | | | | |
| Self-diagnosis – self report of having ever had any of 8 CMD |  |  |  |  |  |  |  |  |  |  |  |  |
| yes | 6.46 [5.14, 8.12] | <.0001 | 3.20 [2.37, 4.31] | <.0001 | 15.99 [10.58, 24.16] | <.0001 | 2.22 [1.24, 3.98] | .008 | 2.48 [1.56, 3.93] | <.0001 | .71 [.38, 1.33] | .28 |
| no (ref) |  |  |  |  |  |  |  |  |  |  |  |  |
| Prof diagnosis – ever diagnosed with any of 8 CMD |  |  |  |  |  |  |  |  |  |  |  |  |
| yes | 4.78 [3.98, 5.76] | <.0001 | 1.75 [1.36, 2.25] | <.0001 | 14.83 [10.91, 20.16] | <.0001 | 3.37 [2.14, 5.31] | <.0001 | 3.10 [2.20, 4.37] | <.0001 | 2.02 [1.25, 3.26] | .004 |
| no (ref) |  |  |  |  |  |  |  |  |  |  |  |  |
| Ever admitted to hospital or ward specialising in mental health |  |  |  |  |  |  |  |  |  |  |  |  |
| yes | 2.42 [1.37, 4.26] | .002 | 1.02 [.56, 1.86] | .95 | 21.01 [14.13, 31.25] | <.0001 | 6.43 [3.91, 10.56] | <.0001 | 8.70 [5.02, 15.07] | <.0001 | 6.11 [3.40, 10.98] | <.0001 |
| no (ref) |  |  |  |  |  |  |  |  |  |  |  |  |
| Life Experiences | | | | | | | | | | | | |
| Childhood Adversity | 1.54 [1.42, 1.67] | <.0001 | 1.22 [1.12, 1.34] | <.0001 | 2.13 [1.95, 2.33] | <.0001 | 1.53 [1.38, 1.69] | <.0001 | 1.39 [1.27, 1.52] | <.0001 | 1.25 [1.12, 1.39] | <.0001 |
| Trauma | 1.29 [1.25, 1.33] | <.0001 | 1.16 [1.12, 1.21] | <.0001 | 1.52 [1.46, 1.58] | <.0001 | 1.24 [1.18, 1.30] | <.0001 | 1.18 [1.13, 1.23] | <.0001 | 1.06 [1.004, 1.12] | .04 |
| Social Support Score | .94 [.91, .97] | <.0001 | .94 [.90, .99] | .008 | .89 [.86, .92] | <.0001 | .96 [.91, 1.02] | .18 | .95 [.91, .98] | .005 | 1.02 [.97, 1.07] | .53 |

*Reference category: no suicidal history

**Reference category: suicidal thoughts

Appendix 8 – Multinomial logistic regression of demographic characteristics, health and psychosocial factors associated with suicidal history group membership in males

| Full Ideation to Action Model in Males | | | | | | | | | | | | |
| --- | --- | --- | --- | --- | --- | --- | --- | --- | --- | --- | --- | --- |
| Model Variables | **Suicidal Ideation vs No Suicidal History*** | | | | **Suicide Attempts vs No Suicidal History*** | | | | **Suicidal Thoughts vs Suicide Attempts**** | | | |
|  | **Unadjusted OR** | **P value** | **Fully Adjusted OR** | **P value** | **Unadjusted OR** | **P value** | **Fully Adjusted OR** | **P value** | **Unadjusted OR** | **P value** | **Fully Adjusted OR** | **P value** |
| Sociodemographics | | | | | | | | | | | | |
| Age | .76 [.67, .85] | <.0001 | .88 [.74, 1.06] | .18 | .69 [.57, .82] | <.0001 | .57 [.45, .73] | <.0001 | .91 [.74, 1.12] | .36 | - | - |
| Marital Status |  |  |  |  |  |  |  |  |  |  |  |  |
| Same-sex couple | - | - | - | - | - | - | - | - | - | - | - | - |
| Divorced or separated | 2.28 [1.64, 3.16] | <.0001 | 1.40 [.97, 2.02] | .07 | 2.53 [1.53, 4.17] | <.0001 | .73 [.44, 1.22] | .23 | 1.22 [.68, 2.19] | .50 | .68 [.38, 1.23] | .20 |
| Widowed | .71 [.41, 1.24] | .23 | 1.17 [.66, 2.08] | .59 | .93 [.41, 2.11] | .87 | 1.19 [.55, 2.55] | .66 | 1.52 [.54, 4.22] | .43 | .91 [.34, 2.43] | .85 |
| Single | 2.29 [1.78, 2.95] | <.0001 | 1.84 [1.36, 2.48] | <.0001 | 4.29 [3.02, 6.09] | <.0001 | 1.57 [1.05, 2.36] | .03 | 1.72 [1.12, 2.65] | .01 | 1.14 [.73, 1.79] | .56 |
| Married or cohabitating (ref) |  |  |  |  |  |  |  |  |  |  |  |  |
| Ethnicity |  |  |  |  |  |  |  |  |  |  |  |  |
| mixed/multiple ethnicities/other ethnic groups | .55 [.20, 1.54] | .26 | .79 [.29, 2.13] | .64 | .68 [.16, 2.81] | .59 | 1.09 [.34, 3.45] | .89 | 1.22 [.22, 6.76] | .82 | - | - |
| Asian/Asian British | .36 [.17, .77] | .009 | .73 [.37, 1.46] | .38 | .88 [.40, 1.91] | .74 | 1.62 [.74, 3.58] | .23 | 2.45 [.84, 7.11] | .10 | - | - |
| black/African/Caribbean/black British | 1.03 [.48, 2.17] | .95 | .98 [.43, 2.27] | .97 | 1.26 [.45, 3.51] | .67 | .76 [.27, 2.14] | .61 | 1.22 [.36, 4.13] | .75 | - | - |
| white (ref) |  |  |  |  |  |  |  |  |  |  |  |  |
| Education |  |  |  |  |  |  |  |  |  |  |  |  |
| no qualifications | .32 [.12, .90] | .03 | .48 [.19, 1.21] | .12 | 1.79 [.66, 4.85] | .25 | 1.63 [.62, 4.26] | .32 | 5.54 [1.38, 22.27] | .02 | 3.28 [.86, 12.44] | .08 |
| Below degree level qualifications | .89 [.67, 1.16] | .40 | .75 [.57, 1.00] | .05 | 1.58 [.98, 2.55] | .06 | 1.00 [.65, 1.54] | 1.00 | 1.76 [1.04, 2.99] | .04 | 1.36 [.82, 2.24] | .24 |
| degree level qualification (ref) |  |  |  |  |  |  |  |  |  |  |  |  |
| Employment |  |  |  |  |  |  |  |  |  |  |  |  |
| economically inactive | .74 [.58, .93] | .01 | .74 [.54, 1.00] | .05 | 1.81 [1.28, 2.55] | .001 | 1.32 [.88, 1.98] | .18 | 2.45 [1.63, 3.68] | <.0001 | 1.21 [.76, 1.94] | .41 |
| unemployed | 1.24 [.69, 2.22] | .48 | .62 [.32, 1.20] | .15 | 3.25 [1.60, 6.60] | .001 | 1.03 [.49, 2.17] | .93 | 2.63 [1.11, 6.22] | .03 | 1.51 [.64, 3.59] | .35 |
| In employment (ref) |  |  |  |  |  |  |  |  |  |  |  |  |
| QIMD |  |  |  |  |  |  |  |  |  |  |  |  |
| 34.17 -> 87.80 most deprived | 1.60 [1.10, 2.32] | .01 | 1.00 [.66, 1.52] | .99 | 4.45 [2.55, 7.79] | <.0001 | 1.56 [.92, 2.65] | .10 | 2.79 [1.46, 5.34] | .002 | 1.59 [.83, 3.06] | .16 |
| 21.35 -> 34.17 | 1.77 [1.23, 2.53] | .002 | 1.25 [.85, 1.82] | .25 | 2.40 [1.31, 4.38] | .005 | .89 [.52, 1.54] | .68 | 1.36 [.69, 2.68] | .38 | .92 [.47, 1.77] | .79 |
| 13.79->21.35 | 1.32 [.91, 1.92] | .14 | 1.10 [.75, 1.60] | .63 | 1.92 [1.04, 3.54] | .04 | 1.03 [.61, 1.74] | .92 | 1.45 [.72, 2.91] | .30 | 1.06 [.55, 2.03] | .87 |
| 8.49 -> 13.79 | 1.55 [1.09, 2.22] | .02 | 1.38 [.97, 1.98] | .08 | 1.13 [.57, 2.23] | .73 | .92 [.53, 1.59] | .77 | .73 [.34, 1.54] | .40 | .71 [.36, 1.39] | .31 |
| 0.53 -> 8.49 least deprived (ref) |  |  |  |  |  |  |  |  |  |  |  |  |
| Rurality |  |  |  |  |  |  |  |  |  |  |  |  |
| village, hamlet and isolated dwellings | .71 [.48, 1.07] | .10 | .87 [.58, 1.30] | .49 | .39 [.18, .83] | .02 | .99 [.49, 1.63] | .72 | .54 [.23, 1.27] | .16 | .94 [.44, 1.99] | .87 |
| town & fringe | .92 [.65, 1.32] | .65 | .95 [.65, 1.39] | .80 | .79 [.46, 1.37] | .41 | .97 [.58, 1.64] | .91 | .86 [.46, 1.62] | .64 | .95 [.51, 1.79] | .88 |
| urban (ref) |  |  |  |  |  |  |  |  |  |  |  |  |
| Health | | | | | | | | | | | | |
| Health in general (SF1) | .79 [.72, .87] | <.0001 | .89 [.79, 1.01] | .07 | .53 [.46, .61] | <.0001 | .85 [.73, .99] | .04 | .67 [.57, .79] | <.0001 | .97 [.80, 1.17] | .76 |
| Multimorbidity | 1.11 [1.06, 1.16] | <.0001 | 1.00 [.94, 1.07] | .99 | 1.25 [1.17, 1.33] | <.0001 | 1.11 [1.02, 1.20] | .02 | 1.13 [1.04, 1.21] | .002 | 1.06 [.97, 1.16] | .22 |
| Smoking History |  |  |  |  |  |  |  |  |  |  |  |  |
| ever smoked | .68 [.53, .87] | .003 | .91 [.70, 1.17] | .44 | .45 [.29, .69] | <.0001 |  |  | .66 [.41, 1.07] | .09 | 1.01 [.64, 1.58] | .98 |
| never smoked (ref) |  |  |  |  |  |  |  |  |  |  |  |  |
| Mental Health and Wellbeing | | | | | | | | | | | | |
| Self-diagnosis – self report of having ever had any of 8 CMD |  |  |  |  |  |  |  |  |  |  |  |  |
| yes | 7.15 [5.53, 9.25] | <.0001 | 2.42 [1.77, 3.31] | <.0001 | 14.31 [8.94, 22.88] | <.0001 | .84 [.49, 1.43] | .51 | 2.00 [1.19, 3.37] | .009 | .43 [.22, .84] | .01 |
| no (ref) |  |  |  |  |  |  |  |  |  |  |  |  |
| Prof diagnosis – ever diagnosed with any of 8 CMD |  |  |  |  |  |  |  |  |  |  |  |  |
| yes | 6.46 [5.10, 8.20] | <.0001 | 3.06 [2.22, 4.23] | <.0001 | 20.25 [13.54, 30.28] | <.0001 | 9.62 [5.67, 16.32] | <.0001 | 3.13 [2.02, 4.87] | <.0001 | 2.72 [1.48, 5.00] | .001 |
| no (ref) |  |  |  |  |  |  |  |  |  |  |  |  |
| Ever admitted to hospital or ward specialising in mental health |  |  |  |  |  |  |  |  |  |  |  |  |
| yes | 4.20 [2.30, 7.66] | <.0001 | .72 [.05, 11.19] | .81 | 23.64 [13.99, 39.97] | <.0001 | 139.59 [37.04, 526.04] | <.0001 | 5.64 [3.02, 10.51] | <.0001 | .79 [.32, 1.97] | .62 |
| no (ref) |  |  |  |  |  |  |  |  |  |  |  |  |
| Life Experiences | | | | | | | | | | | | |
| Childhood Adversity | 1.49 [1.35, 1.66] | <.0001 | 1.16 [1.02, 1.32] | .02 | 2.02 [1.78, 2.29] | <.0001 | 1.75 [1.52, 2.02] | <.0001 | 1.35 [1.18, 1.55] | <.0001 | 1.28 [1.10, 1.49] | .001 |
| Trauma | 1.29 [1.24, 1.34] | <.0001 | 1.16 [1.10, 1.21] | <.0001 | 1.44 [1.37, 1.52] | <.0001 | 1.23 [1.16, 1.31] | <.0001 | 1.12 [1.06, 1.19] | <.0001 | 1.03 [.97, 1.10] | .36 |
| Social Support Score | .93 [.89, .97] | <.0001 | .996 [.94, 1.05] | .87 | .83 [.79, .86] | <.0001 | .81 [.77, .86] | <.0001 | .90 [.85, .93] | <.0001 | .91 [.86, .96] | .001 |

*Reference category: no suicidal history

**Reference category: suicidal thoughts

Appendix 9 – Post Hoc Analysis of Sex Differences in Childhood Adversity and Trauma

In response to a comment from a reviewer, we conducted the following post-hoc analysis. In the whole sample we conducted a series of univariate logistic regressions to investigate whether any variables were significantly overrepresented in men or women (odds ratio associated with men or women). If such differences were identified these variables were incorporated into two composite variables, one for childhood adversity and trauma that were more common in men, and one for childhood adversity and trauma that were more common in women. For the childhood adversity and trauma composite variable in men 12 variables were included and for women 5 variables were combined. These analyses address a limitation identified by (Devries et al., 2013) that there is often a lack of adjustment for confounding variables, such as common risk factors.

**Table 1.** Univariate Logistic Regression Investigating Sex Differences in Childhood Adversity (before 18)

| Childhood Adversity  (Before 18) | Males | | Females | | Sex Differences Univariate Logistic Regression* | |
| --- | --- | --- | --- | --- | --- | --- |
|  | Yes | No | Yes | No | Unadjusted OR | P value |
| Adult in your life hit, beat, physically hurt you (other than smacking) | 431 (48.1%) | 2627 (39.5%) | 465 (51.9%) | 4023 (60.5%) | 1.42 [1.23, 1.63] | <.0001 |
| Got scared or felt really bad because adult in your life called you names, said mean things to you, or said they didn’t want you | 251 (33.6%) | 2807 (41.3%) | 497 (66.4%) | 3991 (58.7%) | .72 [.61, .84] | <.0001 |
| Parent took, kept, or hid you to stop you being with another parent | 71 (34.0%) | 2987 (40.7%) | 138 (66.0%) | 4350 (59.3%) | .75 [.56, 1.00] | .05 |
| Adult in your life shook you very hard or shoved you against a wall or a piece of furniture | 185 (40.7%) | 2873 (40.5%) | 270 (59.3%) | 4218 (59.5%) | 1.01 [.83, 1.22] | .95 |

*reference category: female

**Table 2.** Univariate Logistic Regression Investigating Sex Differences in Childhood Adversity (before 12)

| Childhood Adversity  (Before 12) | Males | | Females | | Sex Differences Univariate Logistic Regression* | |
| --- | --- | --- | --- | --- | --- | --- |
|  | Yes | No | Yes | No | Unadjusted OR | P value |
| Expected to do your own laundry | 929 (37.8%) | 2129 (41.8%) | 1528 (62.2%) | 2960 (58.2%) | .85 [.77, .93] | .85 |
| Had regular dental check ups (reverse scored) | 345 (41.5%) | 2713 (40.4%) | 487 (58.5%) | 4001 (59.6%) | 1.05 [.90, 1.21] | .56 |
| Went to school in clothes that were dirty, torn, didn’t fit because no clean ones available | 688 (46.2%) | 2370 (39.1%) | 802 (53.8%) | 3686 (60.9%) | 1.33 [1.19, 1.50] | <.0001 |
| Went hungry because no one got your meals ready or there was no food in the home | 536 (46.4%) | 618 (53.6%) | 2522 (39.5%) | 3870 (60.5%) | 1.33 [1.17, 1.51] | <.0001 |
| Looked after younger siblings while parents were out | 894 (42.0%) | 2164 (39.9%) | 1234 (58.0%) | 3254 (60.1%) | 1.09 [.98, 1.21] | 1.00 |
| Were ill but no one looked after you or took you to doctor | 410 (43.3%) | 2648 (40.1%) | 536 (56.7%) | 3952 (59.9%) | 1.14 [1.00, 1.31] | .06 |
| Did not have a safe place to stay | 449 (42.4%) | 2609 (40.2%) | 611 (57.6%) | 3877 (59.8%) | 1.09 [.96, 1.25] | .19 |

*reference category: female

**Table 3.** Univariate Logistic Regression Investigating Sex Differences in Trauma

| Trauma | Males | | Females | | Sex Differences Univariate Logistic Regression* | |
| --- | --- | --- | --- | --- | --- | --- |
|  | Yes | No | Yes | No | Unadjusted OR | P value |
| Experienced serious illness or injury at any time in your life | 986 (48.2%) | 2064 (37.7%) | 1061 (51.8%) | 3408 (62.3%) | 1.54 [1.39, 1.70] | <.0001 |
| Experienced serious assault to yourself at any time in your life | 245 (46.6%) | 2805 (40.1%) | 281 (53.4%) | 4188 (59.9%) | 1.30 [1.09, 1.56] | .003 |
| Experienced serious illness or injury to a close relative at any time in your life | 998 (39.8%) | 2052 (40.9%) | 1508 (60.2%) | 2961 (59.1%) | .96 [.87, 1.06] | .96 |
| Experienced serious assault of a close relative at any time in your life | 123 (35.7%) | 2927 (40.8%) | 222 (64.3%) | 4247 (59.2%) | .81 [.64, 1.01] | .06 |
| Experienced death of an immediate family member at any time in your life | 1945 (40.2%) | 1105 (41.3%) | 2899 (59.8%) | 1570 (58.7%) | .96 [.87, 1.06] | .38 |
| Experienced death of a close family friend or other relative at any time in your life | 2094 (39.8%) | 956 (42.2%) | 3161 (60.2%) | 1308 (57.8%) | .91 [.83, 1.01] | .07 |
| Experienced separation due to marital difficulties, divorce or steady relationship breakdown at any time in your life | 883 (36.8%) | 2167 (42.3%) | 1516 (63.2%) | 2953 (57.7%) | .80 [.72, .88] | <.0001 |
| Experienced serious problem with a close friend, neighbour or relative at any time in your life | 378 (39.4%) | 2672 (40.7%) | 582 (60.6%) | 3887 (59.3%) | .95 [.82, 1.09] | .44 |
| Experienced being made redundant or sacked from your job at any time in your life | 1337 (55.6%) | 1714 (33.5%) | 1069 (44.4%) | 3400 (66.5%) | 2.49 [2.25, 2.74] | <.0001 |
| Experienced looking for work without success for more than 1 month at any time in your life | 947 (53.2%) | 2104 (36.6%) | 832 (46.8%) | 3637 (63.4%) | 1.97 [1.77, 2.20] | <.0001 |
| Experienced major financial crisis, equivalent to loss of 3 months income at any time in your life | 461 (52.2%) | 2590 (39.0%) | 422 (47.8%) | 4047 (61.0%) | 1.71 [1.50, 1.97] | <.0001 |
| Experienced something you valued being lost or stolen at any time in your life | 627 (41.5%) | 2424 (40.3%) | 884 (58.5%) | 3585 (59.7%) | 1.05 [.94, 1.20] | .39 |
| Experienced in trouble with police involving court appearance at any time in your life | 370 (76.9%) | 2681 (38.1%) | 111 (23.1%) | 4358 (61.9%) | 5.43 [4.37, 6.75] | <.0001 |
| Experienced time in prison on remand or serving a sentence at any time in your life | 84 (81.6%) | 2967 (40.0%) | 19 (18.4%) | 4450 (60.0%) | 6.64 [4.03, 10.95] | <.0001 |
| Experienced bullying a any time in your life | 751 (40.9%) | 2300 (40.5%) | 1086 (59.1%) | 3382 (59.5%) | 1.02 [.92, 1.14] | .72 |
| Experienced violence at work at any time in your life | 141 (62.1%) | 2910 (39.9%) | 86 (37.9%) | 4382 (60.1%) | 2.47 [1.89, 3.25] | <.0001 |
| Experienced violence in the home at any time in your life | 151 (22.2%) | 2900 (42.4%) | 529 77.8%) | 3939 (57.6%) | .39 [.32, .47] | <.0001 |
| Experienced sexual abuse at any time in your life | 77 (17.0%) | 2974 (42.1%) | 375 (83.0%) | 4093 (57.9%) | .28 [.22, .36] | <.0001 |
| Experienced being expelled from school at any time in your life | 102 (55.7%) | 2949 (40.2%) | 81 (44.3%) | 4387 (59.8%) | 1.88 [1.40, 2.52] | <.0001 |
| Experienced running away from home at any time in your life | 126 (33.2%) | 2925 (41.0%) | 253 (66.8%) | 4215 (59.0%) | .72 [.58, .90] | .003 |
| Experienced being homeless at any time in your life | 131 (41.9%) | 2920 (40.5%) | 182 (58.1%) | 4286 (59.5%) | 1.06 [.84, 1.33] | .63 |

*reference category: female

References

Devries, K. M., Mak, J. Y., Bacchus, L. J., Child, J. C., Falder, G., Petzold, M., . . . Watts, C. H. (2013). Intimate partner violence and incident depressive symptoms and suicide attempts: a systematic review of longitudinal studies. *PLoS medicine, 10*(5), e1001439.
